# Drug Delivery Technologies for the Treatment of Age‐Related Macular Degeneration

**DOI:** 10.1002/advs.202503212

**Published:** 2025-08-25

**Authors:** J Jesus Rodriguez‐Cruz, Jessica Cutrufello, Michael Lam, Shreya Nallaparaju, Nicholas A. Peppas

**Affiliations:** ^1^ Department of Biomedical Engineering The University of Texas at Austin Austin TX 78712 USA; ^2^ Institute for Biomaterials Drug Delivery, and Regenerative Medicine The University of Texas at Austin Austin TX 78712 USA; ^3^ Department of Computational Biology The Univeristy of Texas at Austin Austin TX 78712 USA; ^4^ Department of Chemical Engineering The University of Texas at Austin Austin TX 78712 USA; ^5^ Division of Molecular Pharmaceutics and Drug Delivery College of Pharmacy The University of Texas at Austin Austin TX 78712 USA; ^6^ Department of Surgery and Perioperative Care Dell Medical School The University of Texas at Austin Austin TX 78712 USA

**Keywords:** age‐related macular degeneration, biosimilars, drug delivery, hydrogels, nanoparticles

## Abstract

Age‐related macular degeneration (AMD) is a progressive and degenerative disease affecting the posterior segment of the eye. It currently affects millions of people worldwide, and the gold standard of treatment has remained unchanged for over 20 years. It consists of intravitreal injections of anti‐vascular endothelial growth factor (Anti‐VEGF), which pose significant challenges to patient compliance primarily due to accessibility issues, fear of injections in the eye, and high cost. In this review, a thorough description of the pathogenesis of AMD and the anatomical barriers is presented that must be considered for the design of newer drug delivery systems for the treatment of AMD. Likewise, a critical evaluation of the most recent research efforts in the literature regarding the treatment of AMD using novel drug delivery technologies is provided. Lastly, currently approved therapeutic agents are reviewed for AMD and provide an insight into the recent surge of biosimilars with an outlook on how future therapeutic approaches to AMD should be developed.

## Graphical abstract

There are multiple therapeutic agents available for the treatment of both dry age‐related macular degeneration (AMD) and wet AMD. It is essential to continue innovation by introducing new drug delivery technologies that can incorporate those along with patient compliance and the accessibility of the technology into the early stages of research and development.

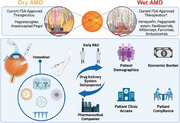

## 1 Introduction

Age‐related macular degeneration (AMD) is an important disease of the eye that remains a major challenge within ophthalmology despite multiple treatments that have been approved by the Food and Drug Administration (FDA) since the early 2000s. AMD has been categorized as a leading cause of blindness in patients over 65 years old, and the World Health Organization has predicted a continuous growth in the cases of this disease, which could affect 240 million patients by 2030.^[^ ^1^ ^]^ Retinal pharmaceutics, likewise, could soon reach and surpass a $22.4 billion market valuation, which is over half of the $42.14 billion global ophthalmic pharmaceutical market projected in 2024.^[^ ^2^ ^]^ Drug delivery of ophthalmic pharmaceutics is therefore strategically positioned at the intersection of biomedical engineering and ophthalmic pharmaceutics development to revolutionize treatment strategies for retinal diseases like AMD, diabetic retinopathy, macular edema, retinitis pigmentosa, and many others.

While there is currently a strong ophthalmic pharmaceutical market, the controlled delivery of drugs to the eye has been a topic of interest for decades. In the early 1970s, with the development and launch of Ocusert, the Alza Corporation introduced the first ophthalmic drug delivery system that was approved by the FDA.^[^ ^3^ ^]^ This device was capable of continuously delivering pilocarpine from ethylene vinyl acetate membranes to the conjunctival sac for the treatment of glaucoma.^[^ ^4^ ^]^ Additionally, the device could be left in the eye for 24 h, and different formulations (Ocusert‐50 or Ocusert‐80) were able to have different pilocarpine release rates to lower intraocular pressure.^[^ ^4^, ^5^ ^]^ While the commercialization of this product was a milestone for drug delivery in ophthalmology, it failed to account for the long‐term patient perspective on comfort with the device, ease of use, and acceptability of new treatments. These critical aspects, often overlooked in ophthalmic drug delivery system development, led to the commercial withdrawal of Ocusert. Therefore, it is imperative for current and future development of these systems to incorporate a holistic approach that weaves in continuous feedback from ophthalmologists and patients into the research and development (R&D) phase.

Accessibility of treatment is another critical aspect that should be prioritized in the design of ophthalmic delivery systems for retinal diseases. For example, to have more accessible treatments for AMD at a global scale, the approximate price of drug delivery systems should be considered even during the early R&D laboratory phase. It is therefore important for researchers to evaluate price differentials between the therapeutic agents already approved for market use. Using these, researchers can design delivery platforms that work with both the expensive and cheap options. This is because, naturally, the use of cheaper therapeutic agents is higher in developing countries and marginalized communities that cannot afford the exorbitant prices of the more expensive ones.

One of the clearest examples of price differentials is found with ranibizumab and bevacizumab, two popular antiangiogenic treatments extensively used for AMD, which will be discussed in depth later in this article. The price difference between these two is significant, with ranibizumab having an average sale price in 2020 of $1716.55.^[^ ^6^ ^]^ Because bevacizumab is used off‐label for the treatment of AMD and must be provided by a compounding pharmacy, it does not have an average sale price reported. However, studies estimate that it is much cheaper than ranibizumab, with a Medicare allowable being less than $100.^[^ ^6^ ^]^ It is fair to assume that even in developed countries like the US, the use of bevacizumab is preferred due to its low price. Indeed, Parikh et.al. conducted a retrospective study from 2006 to 2015 and found that in the first 10 years of antiangiogenic treatment in ophthalmology, bevacizumab was used in 64.6% of injections versus 22% for ranibizumab.^[^ ^7^ ^]^ What is a key lesson about these results is that the price differential can drive acceptability of the treatment, even if one of the therapeutic agents does not have direct approval for AMD from the FDA and is used off‐label as in the case of bevacizumab. Interestingly, the Comparison of Age‐Related Macular Degeneration Treatments Trials (CATT) in 2011 demonstrated that both therapeutic agents can achieve equivalent clinical outcomes in AMD treatment.^[^ ^8^ ^]^ With these results and from the patient's perspective, future AMD drug delivery systems should be designed as tunable platforms that can deliver therapeutic agents of all price ranges for the development of more accessible approaches.

Recent drug delivery research efforts to develop the next generation of treatments for AMD have included intraocular approaches, eyedrop formulations, microneedle patches, and even systemic administration via intravenous injections. In this literature review, we aim to provide drug delivery researchers with a fundamental description of the pathogenesis of AMD. Second, we want to highlight the multiple ocular biological barriers that exist in the delivery of therapeutics to the eye and critically discuss the recent approaches researchers have taken to overcome them. Lastly, we discuss the future of AMD treatments with the introduction of biosimilars into the market and provide insight into the continuous growth of drug delivery approaches for AMD, which provides a plethora of opportunities for novel and better treatments of posterior eye segment diseases.

## 2 AMD Pathogenesis

**Figure 1:**
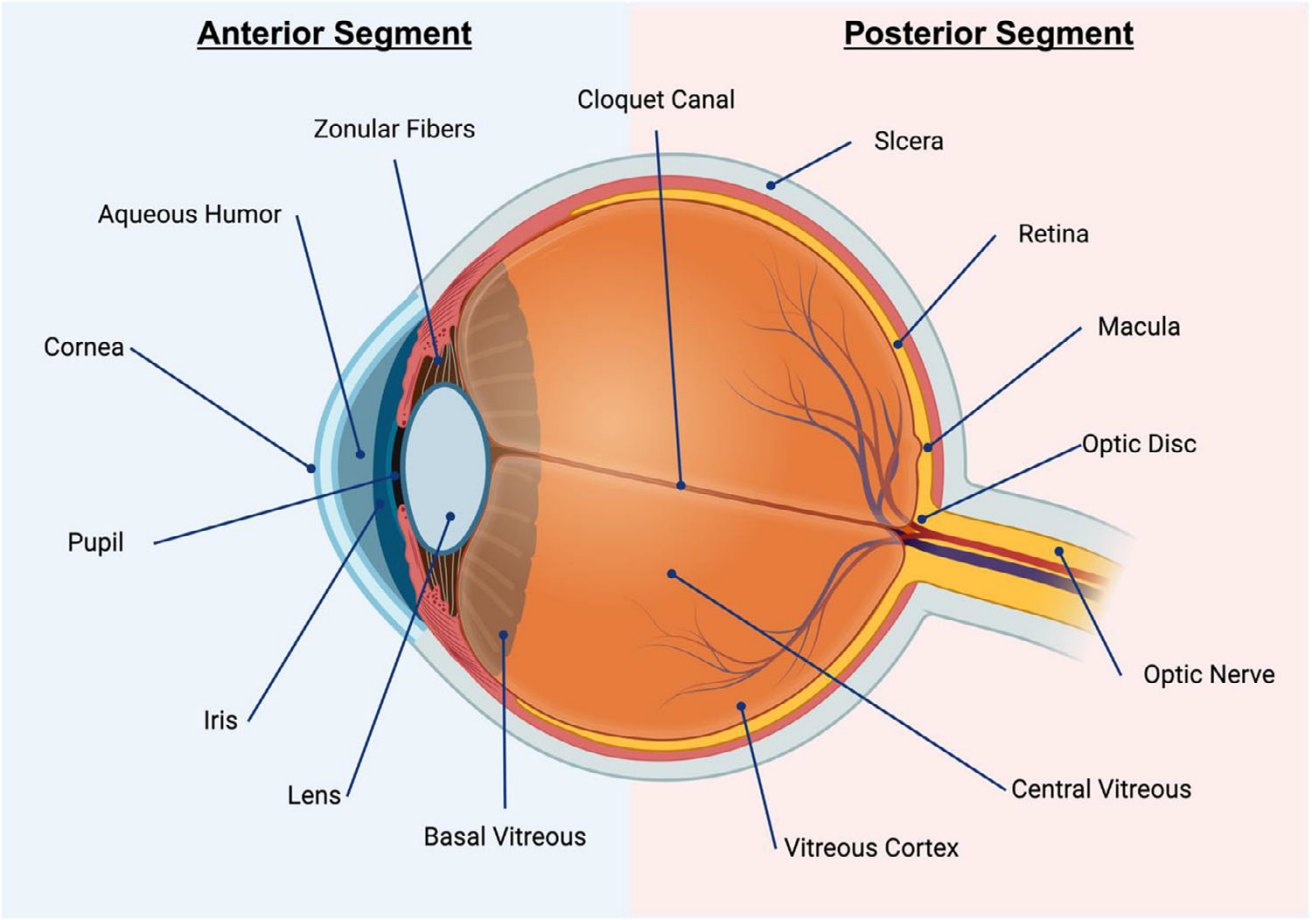
Structures of the ocular anterior and posterior segment.

AMD is a progressive and degenerative retinal disease that can severely affect the macula, a structure located within the retina at the posterior eye segment (**Figure** **1**). This complex structure is critically important to the sharpest and central part of human vision since it houses a high density of cone photoreceptor cells within the fovea and a similarly high density of rod photoreceptors surrounding it.^[^ ^9^ ^]^ The early stages of the disease are characterized by the development of basal drusen structures at the macula, which are mainly composed of lipid and protein deposits.^[^ ^10^ ^]^ These drusen can reach sizes of over 63 µm (at the onset of early AMD) but might develop into larger structures over 125 µm.^[^ ^11^ ^]^ With these latter drusen, the retinal pigment epithelium (RPE) might also present abnormalities in pigmentation as a response to the growth of such drusen.^[^ ^12^ ^]^ Interestingly, however, in these early stages of AMD, the drusen structures do not seem to cause very noticeable changes in the patient's vision. Patients may experience very subtle vision fluctuations, including vision blurring and difficulties seeing in low‐light environments while doing normal everyday activities like reading or driving.^[^ ^13^ ^]^ Therefore, drusen and changes in retinal pigmentation detection at the early stages of AMD is largely limited to a full eye examination by an optometrist or ophthalmologist.^[^ ^10^ ^]^

This early stage of AMD is also known as the dry form, due to the lack of pathological macular neovascularization present. As dry AMD progresses, there can be a gradual manifestation of further vision loss in the form of blind spots or visual scotomas that can last for years. This slow and gradual progression of AMD leads to a late‐stage dry AMD, often referred to as geographic atrophy (GA). While there is still no neovascularization present at this stage, GA is characterized by a critical loss of RPE, photoreceptor cells, and choriocapillaris.^[^ ^14^ ^]^ GA typically results in severe changes to a patient's vision, which impairs their ability to live a normal life, as it typically leads to irreversible blindness. Additionally, the first ethnographic study analyzing the impact of GA in patient's lives by Sivaprasad et al. quantitatively demonstrated that GA has a deep impact beyond daily activities. They demonstrated that GA can alter the life of patients with emotional impact from the fear of going completely blind, financial burden due to the higher costs of visual care, and physical complications due to tripping while walking or even falling.^[^ ^15^ ^]^ While this form of AMD remained treatment‐less for decades, there are now two treatment options that were recently approved by the FDA and which will be discussed thoroughly in a later section (Figure 3).

The neovascular form of the disease, or wet AMD, is the other manifestation of late‐stage AMD. While this form has historically been a lesser percentage of the manifestations of late AMD (i.e more patients develop GA than wet AMD), the neovascular form of AMD is responsible for over 80% of blindness in patients diagnosed with AMD.^[^ ^13^ ^]^ Wet AMD can arise as a rapid progression from the early stages of drusen development mentioned above, and it can take only a matter of weeks to develop. During this timeframe, pathological neovascularization in the retinal space occurs because of increased vascular endothelial growth factor (VEGF) secretion. This VEGF upregulation has been identified as an immune response to the damage within the macula caused by early AMD and it stimulates the rapid growth of new and leaky vasculature at different retinal spaces.^[^ ^16^ ^]^ When fluid leaks at the macula, it causes further severe damage to macular photoreceptor cells and severely impairs a patient's vision. Understanding of the AMD progression is critical to the identification of novel therapeutic targets.

Although a complete picture of the immunosenescent ocular space does not currently exist, recent literature has provided key evidence that as the ocular microenvironment ages, immune‐related events appear to play a central role in the progression of AMD. Indeed, various studies have investigated a variety of these events, including the activation of complement factors and their cascade within retinal tissue, which may play a critical role in both geographic atrophy and neovascularization. Similarly, others have explored how the highly controlled immune environment of the eye can orchestrate the release of cytokines and activation of T cells both in the ocular and extraocular space.^[^ ^17^, ^18^ ^]^ As a more complete picture of the immune system implications in the pathogenesis of AMD is built, more therapeutic targets could be identified, leading to novel treatments and drug delivery systems. Such is the case for the recently approved therapeutic agents for the treatment of dry AMD, which will be discussed in a later section.

## 3 Anatomical Considerations for AMD Drug Delivery System Development

**Figure 2:**
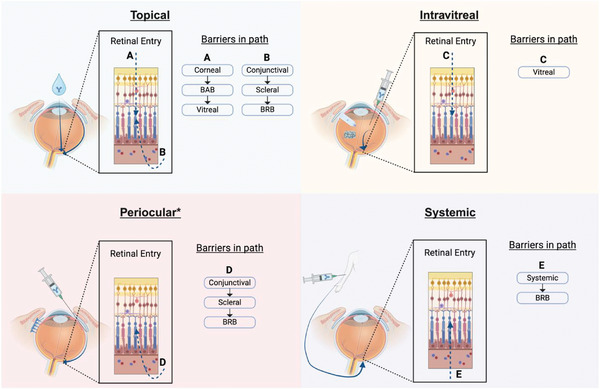
Principal routes of ocular administration and their associated biological barriers in the path to reach the retinal space. *: Periocular routes include injections at the subconjunctival and suprachoroidal space.

The intricate complexity of the eye and AMD makes the design of drug delivery systems for its treatment a critical challenge within the field. The anatomical features of the eye are almost perfectly designed to keep external agents from entering the eye and from penetrating multiple cellular layers within it. While this is of course beneficial for preserving the structural integrity of the eye and its health, it prevents pharmaceutical agents from entering and treating diseases properly (**Table** **1** summarizes challenges and opportunities from this). The main biological barriers to therapeutics entering the eye include the tear film and corneal layers, the blood‐aqueous‐barrier (BAB), the vitreous humor, the conjunctiva and sclera, and the blood‐retinal‐barrier (BRB) (**Figure** **2**).

**Table 1:** Challenges and opportunities for drug delivery approaches from different ocular structures.

Ocular structure	Challenges	Opportunities
Tear film	Amphiphilic character (lipid and aqueous layer) Mucus barrier	Use of amphiphilic carriers that can transport both hydrophilic and hydrophobic cargo Use of mucus‐penetrating nanocarriers or with no mucus interaction (mucus‐inert) to enhance drug absorption into the eye
Cornea	Multi‐cellular layered structure Tightly bound epithelium (presence of tight junctions) Hydrophobic and hydrophilic layers Presence of active transport and metabolism	Actively target the temporary opening of tight junctions for enhanced transcorneal transport (penetration enhancers, protein‐based approaches, etc.) via nanocarrier. Integrate secondary or co‐delivery to the anterior segment by taking advantage of transport at endothelium layer
Vitreous Humor	Aqueous glycosaminoglycan mesh composition Strong anionic character	Potential to form intravitreal drug depots by using cationic carriers and implants Size of the carrier can control diffusion through the mesh
Conjunctiva and Sclera	Presence of both mucus and vascularization (conjunctiva) Stiff collagen matrix (sclera) Scleral architecture plus charge interactions Systemic absorption	Design of carriers can be tailored toward the protection of the drug from systemic absorption (nanocarrier size larger than the vessel pore) Charge interactions with scleral architectural composition hold potential for scleral drug depots acting long term Muco‐inert carriers can enhance drug diffusion through conjunctiva toward the retinal tissues
Blood Ocular Barriers	Presence of tight junctional complexes Molecular weight‐limited transport	Precise control over carrier size and its specificity to ocular vasculature may limit systemic exposure

### 3.1 Tear Film

The tear film is a protective and lubricant layer in the outermost part of the eye. Classic models divide it into three different layers, including a lipid (hydrophobic) layer, an aqueous (hydrophilic) layer, and the mucus layer. The natural amphiphilic structure of the tear film is one of the major barriers to both hydrophilic and hydrophobic compounds entering the eye. Additionally, the mucus layer has an abundance of mucins, secreted by goblet cells in the conjunctiva, that can form a gel barrier (these include MUC 2, 5B, 5AC, and 6).^[^ ^19^, ^20^ ^]^ Currently, the role of the mucus layer of the tear film as a barrier to drug delivery has not been fully studied, and only a few studies have been conducted using small molecular weight molecules, but the results have varied.^[^ ^21^ ^]^ However, more recent studies indicate that engineering nanoparticles to have nearly no interactions with mucus increases drug permeation.^[^ ^22^ ^]^ Regarding AMD, more studies are needed where the mucus layer is altered to examine the permeability of large molecular weight therapeutics (mainly biologics). These types of studies, along with proteomic analysis of the tear film during AMD, will uncover both diagnostic and therapeutic targets. Recently, a proteomic study of the tear film during AMD found upregulation of eight specific proteins that were not previously associated with AMD and could play a key role in monitoring the disease or evaluating novel treatments and their efficacy.^[^ ^23^ ^]^

### 3.2 Cornea

The cornea is a transparent structure of the eye mainly composed of five cellular layers, including the corneal epithelium (in direct contact with the tear film), the Bowman's membrane, the stroma, the Descemet's membrane, and the corneal endothelium.^[^ ^24^ ^]^ Its main physiological function is to refract the light entering the eye, but it also serves as another barrier to bacteria or other things entering the eye due to its unique structural composition.^[^ ^24^ ^]^ Although all layers have important physiological functions, we focus only on the epithelium, stroma, and endothelium as they have unique features that are important to consider when designing drug delivery systems. The corneal epithelium is the main barrier to molecules trying to pass through the cornea, as it is composed of up to seven layers of squamous epithelial cells tightly bound by zonula occludens.^[^ ^25^ ^]^ Thus, it provides a tight barrier to tears and to the paracellular transport of molecules, which directly impacts pharmacokinetics. This presents a unique challenge to the design of drug delivery systems targeting the posterior segment that are administered at the anterior segment, such as topical treatments or drug‐eluting contact lenses. The stroma, which constitutes about 90% of the cornea, is often also considered a barrier to therapeutics permeating through the corneal structures.^[^ ^25^ ^]^ Although not as strong a barrier as the epithelium, it may hinder the permeation of highly hydrophobic drug molecules due to partitioning because of the stromal water composition, which can be over 70% of the total stromal volume.^[^ ^24^, ^26^ ^]^ Lastly, the corneal endothelium is a cell monolayer composed of hexagonal cells with adherens junctions and tight junctions as well as desmosomes.^[^ ^25^, ^27^ ^]^ It is largely responsible for maintaining the cornea hydrated through active transport of molecules and fluids between the aqueous humor and the stromal space.^[^ ^28^, ^29^ ^]^ This highly active transport makes the endothelium a “leaky” barrier despite the presence of cell junctions and is therefore often not considered as a substantial barrier to drugs entering the eye via the trans‐corneal pathway. However, it could be important to consider the transport and highly metabolic state of the corneal endothelium, as it might present opportunities for direct anterior segment delivery of therapeutics.

### 3.3 Vitreous Humor

The vitreous humor is a gel‐like structure in the middle of the eye that is fully transparent and composed of over 90% water along with a polymer matrix of hyaluronic acid and collagen. It provides structural support to the eye and serves as a highway for light and for the diffusion of molecules between the anterior and posterior segments. Similarly, it serves as a barrier to macromolecules and cells that is primarily driven by electrostatic interactions, where anionic particles and molecules have a higher diffusion due to the presence of polyanionic glycosaminoglycans like hyaluronic acid.^[^ ^30^ ^]^ Size restrictions also may play a role in the transport of molecules through the bovine vitreous, with studies showing rapid diffusion of intravitreal PEGylated nanoparticles (neutral charge) of 100–750 nm and 100–200 nm anionic nanoparticles.^[^ ^31^, ^32^ ^]^ While the diffusion of cationic molecules is largely hindered by the strong anionic character of the vitreous humor, researchers have cleverly engineered drug delivery depots that take advantage of this phenomena. Melgar–Asensio et al. recently showed that nanoparticles conjugated with cationic arginine peptides exhibited extended intravitreal presence.^[^ ^33^ ^]^ In a similar manner, Ghosh et al. reported on a unique 97‐amino acid human peptide that binds to the vitreous’ hyaluronan as a way to engineer novel long‐acting anti‐VEGF drugs with three‐ to four‐fold higher half‐life.^[^ ^34^ ^]^ Thus, while the vitreous humor might act as a barrier to drug delivery systems targeting the retinal space, it could also be used as a depot system where drug molecules can be slowly released over time.

### 3.4 Conjunctiva and Sclera

The conjunctiva and sclera constitute most of the ocular surface along with the cornea, and their principal roles include providing structural support and to serve as a barrier to external molecules entering the eye. The conjunctiva is a mucosal layer covering the sclera (bulbar conjunctiva) and the eyelids (palpebral conjunctiva). It is vascularized and contains up to six layers of epithelial cells, which also contain goblet cells that secrete mucins.^[^ ^35^ ^]^ The sclera is a stiff and avascular collagen matrix surrounding the eye, providing structural composition and giving shape to the eye. It is composed of a matrix of elastin and collagen fibers with high surface area and mesh sizes that allow for 10‐fold greater permeability of molecules through the sclera relative to corneal permeation.^[^ ^36^ ^]^ The transscleral transport of macromolecules is mainly affected by molecular weight, charge, and hydrophobicity, with low molecular weight anionic and hydrophilic molecules exhibiting a lower transport lag time.^[^ ^37^, ^38^ ^]^ The conjunctiva also largely limits the transport of drugs into ocular tissues due to its vascularization.^[^ ^39^ ^]^ Systemic drug absorption is the main rate‐limiting factor for drug molecules administered topically, and therefore, critical consideration must be given to conjunctival permeability when designing topical drug delivery systems for posterior segment diseases. Work by Ramsay et al. recently identified permeability values for 32 drug molecules, indicating that surface area, halogen ratio, and hydrogen bond donor were the main factors influencing transport.^[^ ^40^ ^]^ This highlights the importance of molecular aspects determining the permeability of drugs with low molecular weight through the conjunctiva. Further studies are needed to determine such values for protein therapeutics and nanoparticles, which will be critical in the design of newer drug delivery systems.

### 3.5 Blood Ocular Barriers

The blood ocular barriers are not specific structures of the eye but are rather mainly characterized by tight junctional complexes serving as major barriers to molecules entering the eye from systemic circulation. At the anterior segment, the blood‐aqueous barrier (BAB) is comprised of epithelial tight junctions at the iris ciliary blood vessels and at the nonpigmented epithelium.^[^ ^41^ ^]^ Similarly at the posterior segment, tight junction proteins at the retinal pigmented epithelium and the endothelium of retinal capillaries comprise the outer and inner blood‐retinal barrier (BRB), respectively.^[^ ^42^, ^43^ ^]^ Together, both blood barriers highly restrict drug molecules administered systemically from entering the eye, while small molecular weight and lipophilic molecules such as nutrients have been shown to diffuse through the barriers using active forms of transport.^[^ ^44^ ^]^ Other studies investigating size‐dependance of nanoparticles have demonstrated that small 20–50 nm nanocarriers can pass through the BRB after systemic administration.^[^ ^45^, ^46^ ^]^ Due to the low drug concentrations achieved in retinal tissues after systemic administration, high doses of the therapeutic are needed, which might result in toxic side‐effects throughout the body. Additionally, therapeutic agents like biologics have short half‐lives and would need to be protected from non‐specific serum protein interactions to reach the ocular space. Novel drug delivery systems utilizing systemic administration should therefore consider the small size restrictions imposed by the blood ocular barriers and achieve high drug loading along with specific targeting to minimize systemic exposure to the therapeutic.

## 4 AMD Therapeutic Agents in the Clinic

**Figure 3:**
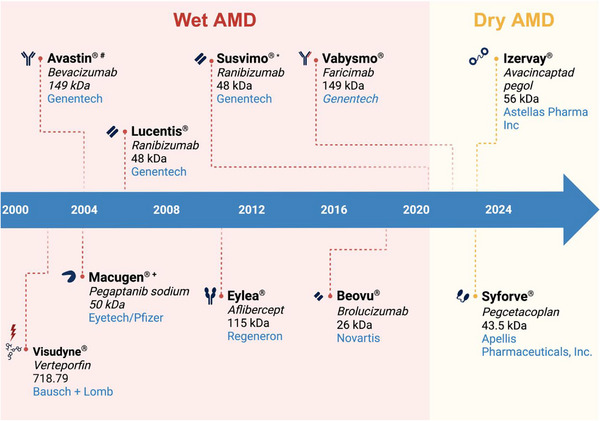
Anti‐VEGF agents in the clinic timeline (not including biosimilars). #: Bevacizumab is not FDA‐approved but largely used “off‐label”. ^+^Was discontinued *: Susvimo was recalled in 2021, but is now back in the market.

In a seminal work by Dr. Judah Folkman published in the New England Journal of Medicine in 1971, the term “anti‐angiogenesis” was introduced and defined as a therapeutic strategy to prevent neovascularization in a tumor implant.^[^ ^47^ ^]^ In further, similarly groundbreaking work by Folkman and Langer, the first factors for inhibition of tumor neovascularization were isolated for the first time from segments of cartilage.^[^ ^48^ ^]^ Although these were fundamental to innovative cancer treatment strategies, it was only a matter of time before the field applied this knowledge in the search for anti‐angiogenic agents for the treatment of AMD, where neovascularization developed as a result of increased expression of VEGF. Currently, these agents are considered the gold‐standard of treatment for the wet form of AMD. Similarly, as the field progressed and the immunological landscape of both forms of AMD was further explored, potential treatments for dry AMD began to appear. Today, only two therapeutic agents for the dry form of AMD have been approved by the FDA and remain the gold standard since their introduction in 2023. Here, we chronologically review the different therapeutic agents available in the clinic for both forms of AMD (see **Figure** **3**).

### 4.1 Wet AMD

#### 4.1.1 Verteporfin

*Verteporfin* (Visudyne) is a photosensitizer used in photodynamic therapy (PDT) for the treatment of neovascular age‐related macular degeneration (AMD) approved by the FDA in 2002. After intravenous administration, it accumulates in choroidal neovascular lesions associated with wet AMD at a dose of 6 mg m^−2^ of body surface area.^[^ ^49^ ^]^ Its peak light absorption is at 689 nm and it can be delivered through a fiber optic, achieving deep penetration into ocular tissues without causing thermal damage.^[^ ^50^, ^51^ ^]^ The PDT molecule also exhibits affinity for low‐density lipoprotein (LDL) receptors, which are typically upregulated in neovascular endothelium at the retina, thus giving it it's high specificity.^[^ ^52^ ^]^ Once accumulated at choroidal vessels, the molecule is activated by light, damages the target cells, and occludes choroidal neovascularization.^[^ ^53^ ^]^ It clears from ocular tissues within 24 h, thereby limiting photosensitivity.^[^ ^54^ ^]^ However, after administration of the treatment, patients must follow protection of both skin and eyes from direct sunlight for several days, which makes it inconvenient.

#### 4.1.2 Bevacizumab

*Bevacizumab* (Avastin) was first approved by the FDA in 2004 for the treatment of colon cancer, with more approvals for other types of cancer since then. However, it is used worldwide for the “off‐label” treatment of wet AMD.^[^ ^55^ ^]^ Bevacizumab is a full immunoglobin G1 antibody that binds all isoforms of VEGF‐A to inhibit choroidal neovascularization.^[^ ^56^ ^]^ Since the results of the CATT trial, its use around the world for the treatment of wet AMD has remained controversial, and no consensus exists around the proper dosage and course of treatment. Due to its large molecular weight (149 kDa), its permeability into the retina is hindered and consequently its clearance is slower than other agents of lower molecular weight.^[^ ^57^ ^]^ While manufactured by the same company as ranibizumab (Genentech), the company has not pursued FDA approval of bevacizumab for the treatment of AMD, and it will most likely continue to be used “off‐label” by ophthalmologists around the world.

#### 4.1.3 Pegaptanib Sodium

*Pegaptanib sodium* (Macugen) received FDA approval for the treatment of wet AMD in 2004, but has now been discontinued by the manufacturer since 2011. It is a 28‐nucleotide RNA aptamer engineered with a covalently bonded polyethylene glycol moiety to promote bioavailability.^[^ ^58^ ^]^ Pegaptanib exhibits low selectivity compared to other anti‐VEGF agents, mainly targeting the heparin‐binding domain of VEGF165, an isoform of VEGF, thereby blocking its interaction with VEGF receptor‐1 and ‐2 tyrosine kinases.^[^ ^58^, ^59^ ^]^ Thus, other agents recently approved are highly superior and bind to most, if not all, isoforms of VEGF.

#### 4.1.4 Ranibizumab

*Ranibizumab* (Lucentis) was first approved by the FDA in 2006 for the treatment of wet AMD. It is a recombinant humanized antigen‐binding fragment (Fab) capable of inhibiting all isoforms of VEGF‐A. Due to its small molecular size (48 kDa) it is able to penetrate the retina faster than other anti‐VEGF molecules like bevacizumab, which has several limitations in penetrating the retina post‐intravitreal injection.^[^ ^60^, ^61^ ^]^ Specifically, Ranibizumab was engineered through phage display‐based affinity maturation to achieve substantially higher binding affinity for VEGF‐A compared to the parent Fab derived from the murine monoclonal antibody A4.6.1.^[^ ^62^ ^]^ The compact Fab fragment design enables enhanced penetration into the retina versus full‐length antibodies following intravitreal delivery.^[^ ^63^ ^]^ Additionally, the lack of an Fc region can prevent inflammatory effects mediated by Fc receptors that could otherwise limit intraocular efficacy.^[^ ^64^ ^]^ Collectively, these optimized design properties allow ranibizumab to potently bind VEGF‐A isoforms and effectively suppress VEGF activity in the retina after injection into the vitreous cavity.

#### 4.1.5 Aflibercept

*Aflibercept* (Eylea) was first approved by the FDA in 2011 for the treatment of wet AMD. It is a fully human recombinant fusion protein composed of the second binding domain of VEGF receptor 1 and the third binding domain of VEGF receptor 2, fused to the Fc region of Immunoglobin G1. Unlike other anti‐VEGF therapies, aflibercept binds and blocks all VEGF‐A isoforms, VEGF‐B, and placental growth factor (PlGF).^[^ ^65^, ^66^ ^]^ Its binding affinity for VEGF (Kd 0.5 pM) is much higher than ranibizumab, bevacizumab, or native VEGF receptors, allowing effective blocking even at low VEGF concentrations.^[^ ^66^ ^]^ The intermediate molecular weight (115 kDa) also enables improved retinal penetration compared to larger antibodies while avoiding rapid clearance of smaller fragments, resulting in an intravitreal half‐life of 7.1 days and duration of action up to 2.5 months, exceeding ranibizumab's 1‐month activity.^[^ ^66^ ^]^

#### 4.1.6 Brolucizumab

*Brolucizumab* (BEOVU) was approved by the FDA in 2019 for the treatment of wet AMD. It is a humanized single‐chain antibody fragment engineered to bind all VEGF‐A isoforms, thereby inhibiting angiogenesis underlying wet AMD pathogenesis.^[^ ^67^, ^68^ ^]^ The very small molecular structure (26 kDa) lacks an Fc region, which allows for both enhanced penetration into retinal and choroidal tissues and increased solubility.^[^ ^69^ ^]^ In vitro analyses demonstrated brolucizumab's substantially higher binding affinity for VEGF‐A and ability to block VEGF receptor activation at markedly lower concentrations compared to other anti‐VEGF biologics.^[^ ^69^, ^70^ ^]^ In a phase 3 clinical trial, brolucizumab achieved better results in regard to best corrected (BCVA) when compared directly with aflibercept.^[^ ^71^ ^]^ Similarly, due to its small molecular size, it can be rapidly cleared from ocular tissues, avoiding possibilities for toxicity and achieving very low levels of inflammation.

#### 4.1.7 Faricimab

*Faricimab* (Vabysmo) is an advanced bi‐specific antibody designed with two recognition sites for angiopoietin‐2 (Ang‐2) and VEGF‐A. This was developed using CrossMAB, which is a technology used in the combination of two antibody domains within one arm of an IgG antibody that is bispecific.^[^ ^72^, ^73^ ^]^ This agent not only aims to mitigate neovascularization by targeting VEGF‐A, but it is also the first to target the angiopoietin/Tie2 signaling pathway. Ang/Tie2 is critical in the stabilization of new vasculature, and studies have shown that inhibition of Ang‐2 results in a reduction of retinal vascular density and reduced vascularization in the center of the retina.^[^ ^74^ ^]^ In recent clinical trials, it was reported that intravitreal faricimab (at a 6 mg dose every 16 weeks) was noninferior to a 2 mg dose of aflibercept every 8 weeks.^[^ ^75^ ^]^ With longer‐acting therapeutic agents and the introduction of potential synergistic therapies of bispecific antibodies, drug delivery design can be significantly improved.

### 4.2 Dry AMD

#### 4.2.1 Pegcetacoplan

*Pegcetacoplan* (Syforve), was the first approved agent for the treatment of geographic atrophy following AMD in 2023. It is a pegylated complement component 3 (C3) inhibitor peptide capable of neutralizing the downstream cascade believed to be critical for GA in dry AMD.^[^ ^76^ ^]^ Additionally, studies have suggested that this form of treatment could also reduce photoreceptor degeneration beyond the already atrophied areas of the macula in dry AMD patients.^[^ ^77^ ^]^ This will be fundamental in the preservation of patient's vision following the treatment. In the FILLY and DERBY/OAKS trials, 15 mg per 0.1 mL monthly or every other month intravitreal dose of pegcetacoplan reduced GA lesion growth compared to controls over a 24‐month period, with an accelerated treatment effect observed between months 18–24.^[^ ^78^ ^]^ Currently, the manufacturer is conducting an extension trial (NCT04770545) to evaluate the long‐term safety and efficacy of the pharmaceutical, which will be critical in the long‐term success of the treatment.

#### 4.2.2 Avacincaptad Pegol

*Avacincaptad pegol* (Izervay) was the second therapeutic approved by the FDA in 2023 for the treatment of geographic atrophy following AMD. It is a complement C5 inhibitor that prevents cleavage of C5, thereby stopping the formation of C5a and C5b fragments involved in inflammation and cell death pathways implicated in AMD progression.^[^ ^79^, ^80^ ^]^ In phase II/III clinical trials, monthly intravitreal administration of avacincaptad pegol resulted in reduced growth rate of geographic atrophy lesions compared to controls.^[^ ^76^ ^]^ As a short and pegylated oligonucleotide sequence, avacincaptad pegol remains in high concentrations in the vitreous humor but in very low plasma concentrations.^[^ ^81^ ^]^ These attributes, along with their biodegradation in case of systemic exposure, prevent possible systemic toxicity, although long‐term studies are needed to assess the dangers of repeated and long‐term administration.

## 5 Recent Advances in AMD Drug Delivery Approaches

The investments in the research pipeline of retinal therapeutics like those used for the treatment of AMD, have grown significantly in the last few years, with billions of dollars put toward the development of newer therapies. Drug delivery as a field is optimally positioned to deliver the next generation of both therapeutic agents and treatment strategies to enhance the current gold standard for both forms of AMD. Researchers continue to develop innovative and complex systems that can deliver therapeutics to the posterior segment in the form of smart eyedrops, injectable nanoparticle and hydrogel drug depots, microneedle technologies, and intravitreal implants, among many others. In this section, we aim to critically review the most recent work in the development of drug delivery technologies for the treatment of AMD.

### 5.1 Topical Approaches

Topical treatment approaches like those in the form of eyedrops, are highly accepted by patients due to their ease of administration, and because they can be self‐administered.^[^ ^82^ ^]^ This is a tremendous advantage over other delivery systems, since problems accessing high‐quality eyecare are a burden to many even in developed countries.^[^ ^83^ ^]^ However, studies have shown that the drugs contained in eyedrops have very poor bioavailability once a drop is instilled at the surface of the eye.^[^ ^84^ ^]^ Due to multiple and complex biological barriers present in the eye, only around 5%–10% of the drug from a 30 µL eyedrop reaches the anterior segment of the eye.^[^ ^85^ ^]^ Therefore, topical approaches have been largely discarded as a treatment alternative for diseases of the posterior segment like AMD. Nonetheless, research efforts in topical approaches are emerging as very promising non‐invasive therapeutic approaches to treat the back of the eye with increased access. This has been achieved in pre‐clinical research by largely focusing on improving the ocular bioavailability of drugs by enhancing the rheological properties of eyedrop formulations, incorporating moieties that enhance penetration of barriers like the corneal and conjunctival epithelium, and by encapsulating therapeutics in nanoparticles and microparticles, among many other approaches described here.

Harnessing the properties of natural polysaccharides to form nanocomplex self‐assembling structures with peptides has recently gained the attention of the field due to their simplicity of fabrication and thus their potential in large‐scale manufacturing. Zhuang Liu and coworkers recently introduced a novel fluorinated chitosan eyedrop that can deliver a variety of therapeutic agents to the retinal space via topical administration.^[^ ^86^ ^]^ Their optimized system, consisting of chitosan grafted with perfluoroalkyl carboxylic acid at a 4.9% fluorocarbon substitution, could form stable nanocomplexes with IgG (at a 1:1 ratio) resulting in sizes around 200 nm.^[^ ^86^ ^]^ Further, the team showed that these nanocarriers exhibited increased permeation through the cornea in Franz diffusion cell *ex vivo* experiments and that high concentrations of IgG could reach the retina of mice and rabbits 6 h after administration during in vivo studies. Their proposed mechanism of delivery indicates that the nanocarriers accumulate at the periocular space and then reach the retina via the conjunctiva‐blood‐retinal pathway. This is most likely due to the carrier's size, which prevents its clearance through the blood vessels present in the conjunctiva. Similarly, the team demonstrated via confocal microscopy and TEER measurements that the fluorinated chitosan was able to temporarily redistribute tight‐junction proteins, which results in increased drug permeation. The mechanism via which this occurred was through altered phosphorylation, which changes the cytoskeleton of cells and results in temporary tight junction disruption. This highlights the large therapeutic potential of directly targeting tight junctions as a method to increase the concentration of drugs entering the eye.

Most recently, their team also introduced another chitosan‐based eyedrop that harnesses the mucus‐penetrating abilities of zwitterionic materials for the treatment of dry AMD.^[^ ^87^ ^]^ Zwitterionic polymers are materials that contain both positive and negative charges but that bear an overall neutral charge, imparting the material with several properties, like mucus‐penetrating abilities.^[^ ^88^ ^]^ Here, they used raft polymerization to synthesize a chitosan‐zwitterionic polymer that could form stable nanocomplexes with a variety of biologics, including adalimumab and catalase, and IgG for optimization of the system. They developed nanocomplex structures of Chitosan (CS) grafted with 2‐(methacryloyloxy)ethyl]dimethyl‐(3‐sulfopropyl) ammonium hydroxide (SBMA) at an optimal ratio of SBMA:CS of 0.5:1. Due to the inherent ability of chitosan to open tight junctions, the nanocomplexes were able to temporarily disrupt both corneal and conjunctival tight junctions, resulting in increased penetration of the therapeutics, achieving reduced dry AMD symptoms in a mice in vivo model. While the long‐term stability of the nanocomplexes and their effectiveness in larger animal models needs to be studied, the team presents a noninvasive approach to deliver relatively high concentrations of therapeutics to the retina.

Triblock copolymers have also been critical in the development of eyedrop formulations aimed at delivering therapeutics to the posterior segment of the eye. Their superior modularity, relative nontoxicity, and ability to self‐assemble into micellar structures enable researchers to engineer approaches to overcome the drug delivery barriers of the topical route of administration. Recently, Zhao et al. developed antiangiogenic micelles composed of poly(ethylene glycol) (PEG), poly(propylene glycol) (PPG), and polycaprolactone (PCL). These were able to achieve a remarkably high aflibercept encapsulation efficiency of 47.3%. As a result, a single drop of aflibercept‐loaded micelles was able to achieve 4‐times higher vitreous humor concentration than the therapeutic by itself in an *ex vivo* porcine model.^[^ ^89^ ^]^ Similarly, the topical treatment achieved reduced levels of retinal vessel leakage in their mice in vivo model. While the molecular mechanism of increased transcorneal and transscleral permeation triggered by the micelles remains unclear, it was suggested that this may be a result of increased transcytosis through the corneal epithelium.

With a slightly different approach, and without disruption of tight junctions, Kim et al. were able to achieve similar results. They reported high retinal concentrations of sunitinib malate and significantly reduced levels of choroidal neovascularization with once‐a‐day dosing of their gelling eyedrop in both a rabbit and a pig in vivo model.^[^ ^82^ ^]^ By fabricating a hypotonic eyedrop formulation of Pluronic F127 (a triblock copolymer of polyethylene oxide‐polypropylene oxide‐polyethylene oxide), they took advantage of the thermosensitive character of the triblock copolymer. Using concentrations below its critical gelling concentration, they ensured the eyedrop remained a liquid In vitro but underwent a sol–gel transition at the surface of the eye to form a uniform film. This gel‐like film at the ocular surface extended the residence time of drugs within the eyedrop and prevented their clearance by tears or blinking. These results were achieved through modification of the physicochemical properties of the eyedrop formulation alone, which suggests it could further be improved by integrating specific cell targeting or tight junction modulators that increase drug concentration at the retina and potentially reduce the dosing regimen.

Sophisticated engineering of nanotechnology also poses a promising approach through which therapeutics could reach the retina after topical administration. The encapsulation or complexation of a therapeutic with a nanocarrier not only protects it from degradation or intraocular clearance, but it also presents a toolbox for increasing concentrations of the drug inside the eye. Sun et al. recently introduced a dual‐modified liposome topical formulation capable of delivering combercept (an anti‐VEGF agent) to the retina of mice. Their approach focused on modifying a liposome carrier with penetratin and hyaluronic acid to both increase drug penetration through ocular barriers and specifically target the retinal pigmented epithelium. Their optimal carriers were stable for up to two weeks and achieved a vitreous humor drug concentration of 18.74 ng mL^−1^ only 4 h after a single topical administration, which is comparable to what other preclinical studies report two days after administering intravitreal injections of the same therapeutic.^[^ ^90^ ^]^ Similarly, Badia and coworkers recently reported on 3 nm anionic cerium oxide nanoparticles as eyedrop formulations for repeated topical administration. Their nontoxic nanocarrier exhibited a significant reduction of choroidal vessel sprouting in mice choroidal explant studies at a 500 µm concentration.^[^ ^91^ ^]^ Notably, their approach did not deliver a protein, but rather relied on the antioxidant properties of the carriers, which resulted in a reduction of inflammatory cytokines, and consequent reduction of choroidal vessel leakage. While no systemic exposure to other body organs was reported, the repeated administration of the nanoparticles should be studied long‐term. Similarly, the exact mechanism of penetration through the ocular barriers should be studied thoroughly in corneal and conjunctival tissues.

Research efforts developing topical treatments for AMD, like the ones described in this section, have elegantly overcome key drug delivery barriers facing topical administration to the eye. This is largely achieved by engineering the materials in their eyedrops and carefully controlling the physicochemical properties of the formulation. Within the next five or ten years, we should see combinatorial approaches where the materials of the eyedrop are engineered to increase drug residence time, and nanocarriers are incorporated to modulate tight junction barriers. Similarly, future studies assessing the molecular mechanisms of nanocarrier and drug transport through the different ocular tissues will provide further avenues for enhancing the efficacy of topical formulations. Thus, newer approaches will exhibit increased transscleral and transcorneal permeation while simultaneously overcoming dynamic and static ocular surface barriers, which would be highly promising in the delivery of therapeutics to the posterior segment via the topical route.

### 5.2 Intraocular Approaches Via Injections

Injectable delivery systems can bypass ocular surface barriers, providing direct delivery to the posterior eye segment. These approaches focus on enhancing the therapeutic delivery via intraocular or periocular injections, which are categorized depending on the delivery site and include intravitreal, subconjunctival, subretinal, and sub‐tenon injections. Indeed, current FDA‐approved therapeutic agents for both forms of AMD are administered in the form of an intravitreal injection, placing the therapeutic directly in the vitreous humor and closer to the retina. However, due to their invasive nature, research efforts are now focused on formulating long‐term systems with sustained drug release over months, which could potentially lower the number of injections a patient would have to get for AMD treatment. Similarly important, these treatments still require administration by a skilled healthcare professional, which may result in a burden for marginalized communities with limited access to high‐quality eyecare.^[^ ^83^ ^]^

#### 5.2.1 Hydrogels

Hydrogels have gained attention in the design of ocular drug delivery systems as they can introduce a variety of advantages, including long‐term controlled release of a single or multiple therapeutic agents, superior biocompatibility, high spatiotemporal control via nanoparticle integration or stimuli‐responsive release, and modularity in their design.^[^ ^92^, ^93^ ^]^ To achieve this within the vitreous humor cavity, subconjunctival space, and suprachoroidal space hydrogels must either form in situ or be injected in a pre‐formed or pre‐crosslinked state. Either form imposes significant engineering requirements on the gelation kinetics, injectability, and dissolution of the system, which can indirectly impact its drug delivery performance once inside the eye. Researchers designing such systems must pay attention to the full characterization of the hydrogel and its release performance In vitro and in vivo (or in silico) for producing high‐quality preclinical products.

Recently, Jiang and coworkers introduced a novel thermosensitive intravitreal hydrogel that combines the therapeutic effects of rapamycin and ranibizumab for a boosted AMD therapy. By targeting the serine/threonine kinase pathway with rapamycin along with the anti‐VEGF capabilities of bevacizumab, their hydrogel depot system tackled autophagic dysfunction, oxidative stress, and neovascularization as a therapy for wet AMD.^[^ ^94^ ^]^ With a single intravitreal administration of the hydrogel (composed of hyaluronic acid and Pluronic F127), the team achieved sustained release of both drugs for up to 14 days. This resulted in drug accumulation at the retina and choroid, which led to a significant reduction of choroidal neovascularization in a mouse model. In a similar approach, the team had previously introduced the thermosensitive intravitreal hydrogel system loaded with a microemulsion of Rhein and Baicalein, which were both decorated with borneol to achieve increased retinal penetration.^[^ ^95^ ^]^ The antiangiogenic and anti‐oxidative properties of the hydrogel system were demonstrated both In vitro and in vivo. Notably, a single intravitreal administration of the system significantly reduced choroidal neovascularization and RPE damage in mice with drug accumulation at the retina for 14 days. Awwad et al. similarly introduced an in situ forming intravitreal hydrogel of poly(N‐isopropylacrylamide) PNIPAAm and acrylated hyaluronic acid as a biodegradable crosslinker. The hydrogel could be loaded with bevacizumab, and In vitro release of the antibody was demonstrated for up to 50 days, preserving its activity and functionality.^[^ ^96^ ^]^ Lastly, Gao et al. recently reported on an injectable supramolecular nanofiber hydrogel system that could exert anti‐oxidant and anti‐angiogenic effects via reactive oxygen species (ROS) scavenging and release of anti‐VEGF. Their system achieved significantly reduced choroidal neovascularization and vessel leakage without side‐effects 4 weeks after intravitreal injection compared to the anti‐VEGF alone.^[^ ^97^ ^]^ Similarly, intravitreal injection of the betamethasone phosphate disodium‐based hydrogel was shown to inhibit the production of several inflammatory factors associated with inflammation.

Injection of drug delivery systems in the subconjunctival and suprachoroidal space is often considered a less invasive approach when compared to intravitreal administration. The potential to inject long‐term releasing hydrogels in this space could significantly lower the number of administrations of the therapy and still achieve therapeutic efficacy. Jung et al. developed an in situ forming hyaluronic acid hydrogel capable of six month anti‐VEGF release that could be injected in the suprachoroidal space. The system consists of a bevacizumab‐hyaluronic acid that is covalently crosslinked 5 s before the injection occurs and that gels in situ within an hour of injection.^[^ ^98^ ^]^ While bevacizumab release occurred for up to eight months In vitro, the hydrogel achieved six month release in vivo with rabbits. Histological assessment of the suprachoroidal space revealed no damage indicative of the biocompatibility of the materials used, but the team did report a temporary decrease in intraocular pressure. In a similar manner, Gade and coworkers recently reported the development and early characterization of an intrascleral chitosan‐PNIPAAm hydrogel for the delivery of sunitinib malate to the retina. Their biocompatible hydrogel approach exhibited antiangiogenic efficacy in *ex vivo* studies and biocompatibility In vitro with ARPE‐19 cells.^[^ ^99^ ^]^ While these results are promising, further characterization of the hydrogel in vivo and long‐term will be crucial for its advancement.

The work discussed in this section presents examples of how single or combinatorial therapy with synergistic effects can be achieved via the use of hydrogels as drug delivery systems for the treatment of AMD. While the results are promising, further studies are needed to evaluate the biodegradability of the hydrogels and their long‐term biocompatibility after repeated dosing in preclinical studies. Similarly, the identification of exact mechanisms of action and full retinal pharmacokinetics after the therapeutic agents are released from the hydrogel will provide significant insight into the performance of the drug in a clinical setting. Together, intravitreal hydrogels present exciting avenues for innovative systems that can deliver drugs to the retina as AMD therapies. With the recent introduction of therapeutics for dry AMD, we should also see a surge in hydrogel systems designed for the treatment of geographic atrophy related to AMD.

#### 5.2.2 Nanocarriers

The incorporation of nanotechnologies in intravitreal drug delivery systems has been an attractive approach in AMD research for decades. The use of such can introduce controlled and stimuli‐responsive release, protection of the therapeutic cargo from intraocular clearance or metabolism, targeting abilities via nanoparticle decoration, and enhanced biodistribution in ocular tissues.^[^ ^100^ ^]^ Properties such as nanoparticle size and stability, loading capacity, biocompatibility with ocular tissues, and release mechanisms should be extensively studied and characterized to produce high‐quality pre‐clinical products. Due to the wide variety of therapeutic agents available in the market, optimal drug delivery platform design for AMD should also consider the use of both low and high molecular weight therapeutics. Ultimately, creating a wide array of well‐characterized drug delivery systems can not only increase their clinical translation but also potentially increase accessibility of the treatments.

Tavakoli et al. recently introduced anionic pegylated liposomes capable of delivering sunitinib, a tyrosine kinase inhibitor that blocks VEGF‐induced signaling pathways. Their 104 nm monodisperse liposomes exhibited high encapsulation efficiency and release of the therapeutic for up to 3 days after intravitreal administration.^[^ ^101^ ^]^ This led them to achieve significant prevention of retinal vessel leakage in mice using their liposome formulation versus the therapeutic alone which did not exhibit any prevention. Similarly, Li and coworkers recently reported successful co‐delivery of mRNA‐150 and quercetin via solid lipid nanoparticles after intravitreal administration. The quercetin acted as a VEGF downregulator by regulation of hypoxia‐inducible factor 1‐alpha and interleukin‐8 and the mRNA‐150 can alleviate angiogenesis by downregulating genes necessary for that process.^[^ ^102^ ^]^ Their 200 nm carriers were modified with an asparagine‐glycine‐arginine peptide to selectively target endothelial cells in retinal vessels and also had high encapsulation efficiencies of both therapeutics (above 85%). The team also demonstrated that carriers effectively targeted the retina and delivered both therapeutics in vivo with mice, resulting in reduced choroidal neovascularization lesion volume. These results are highly important since currently, liposomal formulations are well‐positioned for clinical translation, as recently evidenced by the COVID‐19 vaccine development. However, while these results are promising, further studies are needed to evaluate the long‐term safety of the treatment and its efficacy in larger animal models. Further, the therapeutic agents themselves are not currently FDA‐approved and would also be subject to an extensive and rigorous clinical development before reaching patients. Nonetheless, these research efforts represent unique and novel nanoengineering approaches for overcoming drug delivery barriers in the eye.

Other types of nanocarriers, such as polymeric nanomicelles to encapsulate anti‐VEGF therapeutics are also attractive as these can encapsulate both hydrophilic and hydrophobic cargo. Chang et al. recently developed self‐assembled PLGA nanoparticles loaded with axitinib (AXI) and astaxanthin (AST) as a method to reduce oxidative stress and inflammation in the retina. Their loaded nanocarriers exhibited anionic charge and a size of 114 nm with high encapsulation efficiency for both therapeutics (87.21% for AST and 79.63% for AXI).^[^ ^103^ ^]^ After a single subconjunctival‐injection in mice, the researchers showed the release of both therapeutics was sustained for up to two months and that, further, the therapeutics reached the retina from the subconjunctival space in therapeutically relevant concentrations. This led to observed reduction of retinal vessel leakage and edema achieved by the drug delivery system, along with significant anti‐inflammatory and antioxidant effects. Notably, the team also reported potential visual recovery after treatment with the nanoparticle system, evidenced by recovered photoreceptor function. Thus, this work presents yet another successful example of a combinatorial therapy for AMD treatment. In similar preliminary work, Xu and coworkers recently developed PLGA‐PLA‐based nanomicelles loaded with ranibizumab. Their spherical nanocarriers had monodisperse average size of 26.29 nm, and while the loading capacity of the anti‐VEGF was not reported, only 23.7% loaded ranibizumab was released within 48 h.^[^ ^104^ ^]^ While promising, further studies are needed to evaluate their in vivo fate and long‐term stability after intravitreal administration. Similarly, synthetic high‐density lipoprotein (sHDL) nanoparticles were developed by Mei et al. as a potential intravitreal approach for dry AMD. Incorporation of the particles with rapamycin offers enhanced solubility and reduced lipid deposition via autophagy signaling pathway regulation, though extended safety evaluations are required to study sHDL lipofuscin removal.^[^ ^105^ ^]^ With 10 nm sized nanoparticles capable of 40% encapsulation efficiency, the team demonstrated that their approach offered both rapamycin delivery for anti‐inflammatory effects and the ability to remove cholesterol from target tissues, which may be beneficial in the treatment of drusen rich in lipids. in vivo studies also showed that the nanocarriers reached retinal tissues effectively after intravitreal administration.

Collectively, the research work described in this section provides a glimpse into the grand potential of nanotechnology administered via injections to the eye. Overcoming both the intraocular and ocular surface barriers, these nano‐sized drug delivery vehicles show strong‐preclinical potential for a long‐term releasing therapy that could decrease the number of injections a patient would get for AMD treatment. Further, the research on newer therapeutic targets and the delivery of agents as alternatives to anti‐VEGF molecules via nanoparticles is promising for patients who do not respond well to the anti‐VEGF therapeutics found in the clinic today.

### 5.3 Microneedle Approaches

Regarding the invasiveness of treatments, microneedle technology has introduced less invasive approaches to inject therapeutics into tissues in a rather painless form.^[^ ^106^ ^]^ In ocular drug delivery, these approaches present a unique and novel form of drug delivery to the suprachoroidal and subconjunctival space. Additionally, the complexity and sophistication of the microneedle patch can introduce various aspects beneficial to the delivery of agents into ocular tissues, including long‐term release of therapeutics and facilitating transscleral transport to reach the retina via the choroidal space. Several types of microneedles exist, including solid, hollow, and coated microneedles.^[^ ^107^ ^]^ Although rather new for AMD treatment, microneedles are arising as very promising technologies that could change the administration of anti‐VEGF molecules and other therapeutics to the posterior eye segment.

Recently, Wu et al. reported on protein delivery to the posterior segment of the eye via nanoparticle‐loaded microneedles. The biocompatible PLGA nanoparticles were developed and used to load ovalbumin as a model protein therapeutic to ranibizumab with high encapsulation efficiency.^[^ ^108^ ^]^ By varying sonication time in the nanoparticle fabrication, the researchers achieved different release profiles in vitro, ranging from 8% to 28.5% release of the protein after 77 days. When fabricating the microneedle arrays, the nanoparticles were localized to the tip of the needles, achieving a 24.86 µg ovalbumin encapsulation in the microneedle patch and a daily release of 90 ng from it, which is a therapeutically relevant concentration. *Ex vivo* studies showed that the nanoparticle‐loaded microneedles achieved deep penetration of ovalbumin into the sclera (up to 700 µm). The delivery of high molecular weight molecules from scleral microneedle patches is a significant advancement in the treatment of AMD via microneedles, but the complete pharmacokinetic profile of the molecule once released will be crucial in preclinical work. In a similar approach, Roy and coworkers developed and compared rapidly dissolving scleral and corneal microneedle patches for the delivery of triamcinolone acetonide (TA) to the posterior segment of the eye. The team used polyvinylpyrrolidone and built patches with 25 microneedles, which incorporated a TA nanosuspension.^[^ ^109^ ^]^ in vivo biodistribution of the TA in rabbits after application of both patches revealed that TA was able to reach the retina within 24 h post administration with the scleral patch but no measurable TA was detected at this site using the corneal patch. While both approaches were tolerated by the rabbits in the in vivo study, only the scleral patch would achieve therapeutically relevant concentrations as treatment for AMD.

Although more studies are needed to determine the full potential of microneedles as an alternative and less invasive method of treatment for AMD, the results of the most recent research show grand promise in the delivery of therapeutics (of varying molecular weight) to the retina. Long‐term safety and efficacy of the microneedle arrays should be studied along with the effects of repeated micro‐scale penetration of the scleral tissues. In the next few years, we should see more of these technologies entering the ophthalmology space and creating novel, more accessible treatments for both forms of AMD.

### 5.4 Systemic Approaches

The systemic administration of protein therapeutics in general has been largely limited due to their unstable nature in plasma and short half‐lives.^[^ ^110^ ^]^ Similarly for ocular drug delivery, it has long been considered that the systemic administration of anti‐VEGF therapeutics would be inefficient and could even pose risks of systemic toxicity as high doses would be required to reach the retinal space from systemic circulation. Additionally, as discussed before, the existence of the BRB blocks most molecules from entering the retinal space, thus highly limiting AMD treatment success via systemic administration. Nonetheless, recent approaches have focused on utilizing nanotechnology to extend the half‐lives of protein therapeutics administered systemically.

Recently, Wu and coworkers demonstrated the systemic administration of dendrimer‐peptide conjugates as an innovative approach and alternative to intravitreal administration. Their approach used generation 6 hydroxyl Poly(amidoamine) (PAMAM) dendrimers conjugated to ALG‐1001 peptide via click chemistry.^[^ ^111^ ^]^ This ALG‐1001 peptide has shown good performance in reducing choroidal neovascularization and vascular permeability in mouse models and which showed positive results in a small clinical trial involving 15 subjects with wet AMD.^[^ ^112^ ^]^ After conjugation with the dendrimer, the peptide was resistant to In vitro enzymatic degradation even at high concentrations and could reach target tissues well within its blood circulation time of 4–8 h. The conjugate also exhibited anti‐inflammatory effects and resulted in reduced choroidal neovascularization after 150 µg systemic administration every four days. Work by the same laboratory had previously reported on a similar approach where PAMAM dendrimers were also conjugated to TA and were highly selective for retinal cells, including RPE and injured microglia. Here, they showed that the systemic administration of dendrimers conjugated to TA was able to suppress choroidal neovascularization as a result of reduced levels of inflammation and macrophage infiltration, along with lowering intracellular VEGF levels.^[^ ^113^ ^]^ Xia et al. used a slightly different approach for the systemic administration of therapeutics to treat choroidal neovascularization. They cloaked rapamycin‐loaded PLGA nanoparticles with macrophage cell membranes, ending with anionic and monodisperse 101.3 nm biomimetic carriers.^[^ ^114^ ^]^ The nanoparticles were able to mitigate the overexpression of VEGF‐A by directly interfering with a protein kinase signaling pathway In vitro with ARPE‐19 cells. Daily intravenous administration for 7 days of the cloaked nanocarriers achieved a significant reduction of choroidal neovascularization in mice without concerns for systemic toxicity, demonstrating the safety and efficacy of the approach in vivo.

While dendrimers offer superior potential as systemic nanocarriers for therapeutic proteins due to their small size and biocompatibility, larger‐sized biomimetic nanoparticles have also proven their potential in both targeting the retina after systemic administration and achieving therapeutic effects. These results are promising and constitute alternative delivery strategies for therapeutics targeting the retina. However, intravenous administration still requires an injection and administration by a skilled doctor or nurse. Nonetheless, the emergence of these approaches also represents potential for a more accessible treatment alternative in marginalized communities where a normal clinic is more accessible than high quality eyecare providing intravitreal injections.

## 6 Clinical Status of AMD Treatment

The treatment of AMD at all its disease stages remains a major challenge and a largely unmet clinical need globally. Researchers in both the pharmaceutical and academic sectors are arduously working toward introducing novel therapeutics and treatment strategies for AMD. Several key factors, such as burden to patients, cost, treatment frequency, invasiveness, and effectiveness, not only affect the translation of these experimental treatments into clinics but can also determine their success and superiority over existing treatments. However, before a treatment is ready to be introduced to clinics globally, it must first meet rigorous evaluations by regulatory agencies, which vary by country. While this is a costly and slow process, there are several strategies currently being evaluated with promising preliminary results. In this section, we summarize the clinical status of AMD treatments by critically discussing important and recent clinical trials aimed at FDA approval of new AMD treatments in the US. Lastly, we also discuss the port delivery system as an emerging drug delivery technology, which is already in the clinic.

### 6.1 Clinical Trial Landscape

Over the last few years, there have been many attempts to introduce new treatments for AMD into the clinic. According to the US National Library of Medicine, this is evidenced by over 60 clinical trials that have reached phase 2 and 3 between 2023 and 2025. From new drug candidates to reformulations of existing therapeutic agents and the introduction of alternative delivery routes to intravitreal injections, these clinical trials highlight the arduous attempts to revolutionize AMD treatment.

#### 6.1.1 ABBV‐RGX‐314

One very promising and current case is that of ABBV‐RGX‐314, a gene therapy approach consisting of an adeno‐associated virus serotype 8 vector expressing an anti‐VEGF‐A fragment (antigen‐binding).^[^ ^115^, ^116^ ^]^ This collaboration between the companies REGENXBIO and AbbVie Inc. is both novel regarding the therapeutic agent used and the delivery method, as the clinical trials performed have evaluated both single‐injection suprachoroidal and subretinal delivery for wet AMD. Results from phase one trials were posted in 2023 and published in 2024 demonstrating safety and tolerability of the therapeutic via subretinal injection (NCT03066258).^[^ ^115^ ^]^ These results also provided evidence of potential long‐term VEGF‐A suppression (up to two years). Most recently, efficacy is actively being evaluated with the phase 2 ALTITUDE trial (NCT04567550) as a dose‐escalation study for diabetic retinopathy patients with and without macular edema, and its estimated completion date is set for mid‐2026. One‐year results from this study were shared by REGENXBIO in 2023, indicating that suprachoroidal injection was well tolerated and a single injection at their “dose level 2” showed improvement in disease severity. Two more active trials are underway for this therapeutic agent (NCT05407636 phase 3, and NCT04704921 phase 2b/3) with primary endpoints comparing BCVA relative to patients receiving aflibercept and ranibizumab respectively.

While both suprachoroidal and subretinal injections present advantages over intravitreal injections, like being more precise in the location of administration, they are still invasive. Additionally, these might also become inaccessible to marginalized communities in the future, which have limited access to high quality eyecare. Similarly, the price of these new and advanced therapeutics will also play a key role in the acceptability (by patients) of gene therapy as a novel treatment for AMD.^[^ ^117^ ^]^ Nonetheless, the concept of a single injection treatment for AMD would significantly reduce patient burden and improve treatment compliance which is a major challenge in AMD treatment and one that gene therapy poses to resolve.

#### 6.1.2 CLS‐AX

Another example of an advanced suprachoroidal treatment for AMD in clinical trials was introduced by Clearside Biomedical with CLS‐AX, consisting of Axitinib, a potent tyrosine kinase inhibitor (TKI) capable of blocking 3 VEGF receptors. It is formulated as a suspension with proprietary suprachoroidal microinjection technology. In 2023, the results of a pahse 1/2a dosing escalation study (Oasis ‐ NCT04626128) demonstrated that the suprachoroidal injection was safe and tolerable. Additionally, the study showed early evidence of treatment efficacy, indicated by BCVA stability, and no severe adverse events were observed. This trial was followed by a phase 2b study (Odyssey ‐ NCT05891548), which was completed in 2024. This study showed that wet AMD patients treated with CLS‐AX had stable BCVA, and over 60% of them did not require a second intervention for up to 6 months. These results are encouraging for a TKI‐based therapy formulated as a suspension with the potential to reduce patient burden by dosing every 3‐6 months. The company is reportedly in talks with the FDA as of 2025 to perform phase three pivotal and non‐inferiority studies.

#### 6.1.3 GB‐102

Graybug Vision recently introduced a TKI treatment with a sustained release component as a potential 3–6 month treatment for AMD. GB‐102 is a Sunitinib malate approach that uses PLGA‐based microparticles to form an intravitreal depot formulation.^[^ ^118^ ^]^ Their phase 1 clinical trial (NCT04085341) demonstrated the safety and tolerability of GB‐102 in 2019, but it also showed a mild presence of inflammation and particle presence in the anterior chamber of the eye post treatment. With a new formulation of GB‐102 for improved stability, the company performed a phase 2b study (NCT03953079) to compare the safety of2 GB‐102 doses (1 and 2 mg) with aflibercept treatment. While GB‐102 was well tolerated at 1 mg, the 2 mg dose exhibited many adverse events, such as vitreous humor opacities, and was eventually terminated. Unfortunately, the company does not have a plan as of now to continue with further studies of GB‐102. This highlights the importance of the stability and durability of microparticle formulations intravitreally injected. Nonetheless, it presents a clever depot approach for sustained delivery of intravitreal therapeutics without the need for invasive surgical implantation.

#### 6.1.4 D‐4517.2

The idea of systemic delivery has interestingly long been regarded as non‐practical for AMD applications due to the small vasculature of the eye, which would require subcutaneous injections of very large therapeutic doses for the agent to reach the retinal/choroidal space. However, precision nanomedicine has been posed as a potential solution to this challenge. Indeed, Ashvatta Therapeutics recently introduced D‐4517.2 as a novel hydroxyl‐dendrimer nanocarrier that features a VEGF inhibitor and which can be administered systemically.^[^ ^113^ ^]^ A Phase 1 trial (NCT05105607) with dose escalation proved the safety and tolerability of the therapeutic in the form of a single subcutaneous injection. Currently, a phase 2 (NCT05387837) chronic dosing study is underway to determine pharmacokinetics of D‐4517.2 administered every 2 or 4 weeks for a total of 40 weeks. The company reported preliminary positive results in February of 2025 at the Angiogenesis conference. These included stable BCVA, safety and tolerability of the dosing without adverse effects, and a potential once‐a‐month subcutaneous dosing, which can reduce patient burden.

Subcutaneous approaches for AMD are a critical milestone in the clinical landscape of AMD. These have the potential to improve accessibility since subcutaneous injections are more accessible than IVT injections in marginalized communities. Additionally, subcutaneous administration paired with the precision of the dendrimer nanocarriers has extremely valuable potential of treating both eyes in patients with AMD in both eyes as an alternative to two bilateral IVT injections, which further reduces burden and can increase patient compliance.

All the clinical trials discussed and highlighted in this section serve as evidence of the exciting and increasingly complex clinical status of novel AMD treatments. As work continues, several factors such as cost, accessibility, and FDA approval will largely impact their success in the market. These clinical trials not only mark an unprecedented step toward better AMD clinical care, but they also strengthen the landscape for innovation and the introduction of novel drug delivery technologies for other retinal diseases such as diabetic retinopathy and macular edema. As time progresses, the number of new therapeutic agents and delivery approaches reaching the clinical trial phase will only grow.

### 6.2 The case of the Port Delivery System

The port delivery system is another interesting case in the clinical journey of AMD novel treatments. The PDS is a surgically implantable ocular device capable of continuous delivery of medication into the eye. Once implanted into the sclera, a septum faces the outside of the eye and allows for refilling of a 20 µL reservoir of drug.^[^ ^119^ ^]^ This reservoir is porous and responsible for the control of drug diffusion outside the device and into the vitreous humor of the eye. Genentech announced FDA approval of the PDS loaded with ranibizumab in October of 2021 after successful phase 1–3 clinical trials. The device is capable of up to 6‐month delivery of ranibizumab for the treatment of wet AMD, largely reducing the dosing frequency for patients. However, it is important to note that the company had a recall of the device due to some observed cases of septum dislodgement in 2022, which was resolved last year in April 2024. While the approval of the device is a remarkable drug delivery achievement for wet AMD, it remains highly invasive and may not be available to those communities with limited access to eye care professionals who can implant it or to the implant itself due to its extremely high cost.^[^ ^120^ ^]^

## 7 Biosimilars

As key patents of leading pharmaceutical products for the treatment of AMD, like ranibizumab and bevacizumab expire, biosimilars are increasingly gaining attention as alternative therapeutic agents for AMD, and they have also started to have a stronger presence in the clinic (see **Table** **2**). These are biologic medications intended to replicate FDA‐approved reference products.^[^ ^121^ ^]^ Despite minor structural differences, biosimilars have demonstrated equivalent efficacy, performance, and safety compared to their reference counterparts in a variety of applications.^[^ ^122^ ^]^ From a market standpoint, one of their most promising advantages is their ability to lower treatment cost, making them a more affordable alternative to original biological medications. Indeed, it is currently estimated that biosimilars can save the U.S. healthcare system approximately $100 billion.^[^ ^123^ ^]^ However, studies have suggested that the switch from the original biologic to the biosimilar is highly dependent on a variety of factors, including the ophthalmologists opinions, patient's preferences, and availability.^[^ ^124^ ^]^

**Table 2:** FDA‐approved biosimilars relevant to reference products used in the clinic for AMD treatment.

Reference biologic	Biosimilar product name	Manufacturer of biosimilar	Approval date	FDA indication
Aflibercept (Eylea)	Aflibercept‐ayyh (Pavblu)	Amgen, Inc.	August 2024	Wet AMD Macular edema following retinal vein occlusion Diabetic macular edema Diabetic Retinopathy
	Aflibercept‐jbvf (Yesafili)	Biocon Biologics Inc.	May 2024	Wet AMD Macular edema following retinal vein occlusion Diabetic macular edema Diabetic Retinopathy
	Aflibercept‐yszy (Opuviz)	Biogen Inc. (Manufactured by Samsung Bioepis Co., Ltd.)	May 2024	Wet AMD Macular edema following retinal vein occlusion Diabetic macular edema Diabetic Retinopathy
	Aflibercept‐mrbb (Ahzantive)	Formycon AG	June 2024	Wet AMD Macular edema following retinal vein occlusion Diabetic macular edema Diabetic Retinopathy
	Aflibercept‐abzv (Enzeevu)	Sandoz	August 2024	Wet AMD Macular edema following retinal vein occlusion Diabetic macular edema Diabetic Retinopathy
Ranibizumab (Lucentis)	Ranibizumab‐nuna (Byooviz)	Biogen Inc. (Manufactured by Samsung Bioepis Co., Ltd.)	September 2021	Wet AMD Macular edema following retinal vein occlusion Myopic choroidal neovascularization
	Ranibizumab‐eqrn (Cimerili)	Coherus BioSciences, Inc.	August 2022	Wet AMD Macular edema following retinal vein occlusion Diabetic macular edema Diabetic Retinopathy Myopic choroidal neovascularization
Bevacizumab (Avastin)	Bevacizumab‐awwb (Mvasi)	Amgen, Inc.	September 2017	Various types of cancer (No indication for AMD or other retinal conditions)
	Bevacizumab‐bvzr (Zirabev)	Pfizer Inc.	June 2019	Various types of cancer (No indication for AMD or other retinal conditions)
	Bevacizumab‐maly (Alymsys)	Amneal Pharmaceuticals LLC	April 2022	Various types of cancer (No indication for AMD or other retinal conditions)
	Bevacizumab‐adcd (Vegzelma)	CELLTRION, Inc.	September 2022	Various types of cancer (No indication for AMD or other retinal conditions)
	Bevacizumab‐tnjn (Avzivi)	Bio‐Thera Solutions, Ltd.	December 2023	Various types of cancer (No indication for AMD or other retinal conditions)

Biosimilars are more affordable because they follow a distinct development and regulatory pathway compared to their reference products.^[^ ^125^ ^]^ However, this regulatory pathway is no less rigorous and remains under the strict oversight of the FDA and other regulatory organizations, which review extensive evidence before approving biosimilar applications.^[^ ^126^ ^]^ Unlike the original reference product, however, biosimilar approval emphasizes analytical, comparative clinical, and non‐clinical studies.^[^ ^127^ ^]^ This ensures that the lower development costs of biosimilars do not compromise their manufacturing and production standards, as both original and biosimilar manufacturers must adhere to the same stringent quality manufacturing practices to gain approval.

The reduced costs of medications through biosimilars will provide significant benefits in the treatment cost burden of AMD. Regarding patients, lower prices significantly enhance access to treatments, particularly for those with AMD who often depend on copay assistance programs or social security benefits.^[^ ^128^ ^]^ Even modest cost reductions can make anti‐VEGF agents more affordable for these individuals in marginalized communities. Similarly, decreased costs will also benefit employers, who typically shoulder a substantial portion of healthcare expenses for their employees.^[^ ^129^ ^]^ Savings on medications could then allow employers to allocate healthcare funds toward other valuable services and research efforts toward the development of newer therapies.

The first biosimilar for the treatment of wet AMD in the US was approved by the FDA in 2021. *Ranibizumab‐nuna* (Byooviz) was introduced by Samsung Bioepis as a biosimilar to *ranibizumab* after successful demonstration of long‐term efficacy and safety in a phase III randomized clinical trial. Here, 635 patients completed the trial for 52 weeks, and the biosimilarity of the biologic to the new product was demonstrated with comparable pharmacokinetics.^[^ ^130^ ^]^ Additionally, a post hoc study analyzing the immunogenicity of the biosimilar reported no clinically relevant differences associated with immunogenicity.^[^ ^131^ ^]^ However, despite the positive results, there is still concern among the ophthalmology community regarding the safety of new biosimilars for AMD treatment.^[^ ^132^ ^]^ This further supports the notion that the success and acceptability of biosimilars, although promising and already appearing in the clinic throughout the US, will largely depend on both patients and physicians making the decision to switch from the original product.

Within the next few years, the landscape of biosimilars and their impact on the treatment of retinal diseases like AMD will be better understood and will provide the long‐term and real‐world data that both physicians and patients need to remedy any uncertainty about their safety and efficacy. Similarly, the design of newer drug delivery systems will likely grow to develop newer technologies that can load and release the biosimilar molecules as more of them are approved. It is important to note that the success in the market of drug delivery systems is also dependent on the acceptability of the treatment by patients. Nonetheless, the introduction of biosimilars in the AMD market space will also introduce yet another generation of drug‐delivering technologies with increased potential to further reduce AMD treatment cost.

## 8 Conclusion and Outlook

A growth in the number of researchers throughout the world working in retinal diseases is expected, and the treatment strategies for many of these diseases will incorporate and bring together cutting‐edge innovations in smart biomaterials, novel therapeutic agents, and advanced technologies for drug discovery. As the aging population continues to grow, the demand for therapeutics for retinal diseases will continue to increase and will push innovation in drug delivery technologies. Researchers must thoroughly evaluate the design of ocular drug delivery systems to increase treatment accessibility and reduce invasiveness, increase patient compliance, and consider AMD demographics from the start of the research and development process. For example, a recent study on the disparities of AMD disease burden reported that the prevalence and years lived with the disease were higher in females than males.^[^ ^133^ ^]^ These results also suggest that the biological sex differences in the anatomy of the ocular tissues can also be studied, and these findings should be incorporated into the design of new drug delivery systems for AMD. Accounting for such key biological differences will further enhance the design of the drug delivery technologies and could be key in facilitating the delivery of therapeutics to the posterior segment.

Emerging therapeutics for AMD have already seen large investments by the private pharmaceutical industry sector and will only continue to grow in their journey to the clinic. Bi‐specific antibodies, for example, example of a critical area of research and development for new AMD treatments. Their double mechanism of action opens the door for more efficient treatments and higher control of AMD pathology. Vabysmo (Genentech), which is discussed in this article, already has commercial evidence and excellent clinical trial success as a bispecific antibody treatment in the market. Others in the clinical trial phase include sozinibercept (Opthea), AG‐73305 (Allgenesis Biotherapeutics), and MK‐3000 (restoret, EyeBio/Merck), which is a trispecific approach.

Gene therapy similarly presents an exciting investment opportunity to revolutionize AMD treatment, and some recent agents in the clinical trial pipeline include ABBV‐RGX‐314 (AbbVie and REGENXBIO) and ixo‐vec (Adverum Biotechnologies). These have the potential to create mini‐factories once administered, which could largely reduce dosing frequency. Integrating these with drug delivery technologies that offer controlled release and specificity will also create advanced therapeutic strategies with potential for synergistic therapy, as other medicines could be co‐delivered with the bi‐specific antibodies.

With all systems having key advantages and disadvantages on their own, the field of retinal drug delivery systems is currently experiencing an unparalleled growth, drawing the attention of researchers worldwide. As the market has observed, not all patients respond well to some of the therapeutic agents already in the market for AMD, and some must look for alternatives. Similarly, it can be expected that not all drug delivery technologies will work on all patients, but this suggests that the best system will be the most adaptable and the one that can be tuned to load and release different therapeutic agents regardless of their different physicochemical properties. Thus, tunability of drug delivery systems is a critical property and one that will enable the future of personalized treatments for AMD patients.

Within the next few years, we will see a breadth of companies increasing their R&D efforts and investments while acquiring other companies to grow their pipelines and bring the research performed in labs around the world into the clinic. Developing a sustained drug delivery approach to treat AMD that is accessible to patients, remains non‐invasive, and retains the efficacy observed with the current gold‐standard should be the goal of current research efforts. In the future, this will open the door to further clinically approved drug delivery approaches for other retinal diseases like diabetic retinopathy, retinopathy of prematurity, and macular edema, among many others. Ultimately, these efforts will have a profound impact on the vision of patients across the globe.

## Conflict of Interest

The authors declare no conflict of interest.
